# Psychosocial factors related with caregiver burden among families of children with chronic conditions

**DOI:** 10.1186/s13030-019-0147-2

**Published:** 2019-03-08

**Authors:** Filiberto Toledano-Toledano, Miriam Teresa Domínguez-Guedea

**Affiliations:** 1 Evidence-Based Medicine Research Unit, Children’s Hospital of Mexico Federico Gómez, National Institute of Health, Mexico. Dr. Márquez 162, Col. Doctores, Del. Cuauhtémoc, C.P. 06720 Mexico City, Mexico; 2 Department of Psychology and Communication Sciences, University of Sonora. Blvd. Luis Encinas y Rosales, Col. Centro S/N, 83000 Hermosillo, Sonora Mexico

**Keywords:** Caregiver burden, Family caregivers, Family support, Psychosocial factors, Family functioning, Well-being, Pediatric chronic diseases, Sociodemographic variables, Social support networks

## Abstract

**Background:**

The impact of looking after children who live with complex chronic conditions is a growing public health issue. However, it is unclear whether sociodemographic and psychosocial variables can be used to predict the burden on the caregiver and how the profiles of families of children with chronic diseases are defined and structured. The objective of this study was to identify multivariate sociodemographic and psychosocial variables as well as sociocultural and familial factors to analyze the caregiver burden of family caregivers of children with chronic diseases.

**Methods:**

A cross-sectional study was conducted involving 416 family caregivers of children with chronic diseases at the National Institute of Health in Mexico City. The participants responded to a questionnaire on sociodemographic variables and a battery of 7 instruments that examined caregiver burden, family support, parental stress, anxiety, support networks, family functioning, historic-psycho-socio-cultural premises and the World Health Organization Well-Being Index*.*

**Results:**

A multivariate analysis using hierarchical multiple regression models showed that the variables included in the psychosocial and sociodemographic profile as a whole explained 40% of the variance in caregiver burden, taking sociocultural historical premises, stressors and anxiety into account as positive individual predictors. Negative individual predictors for caregiver burden included upper secondary education, social support networks, family support, family functioning and well-being. The sociodemographic profiles of family caregivers were as follows: female (81.7%); mean age, 31.7 years (standard deviation [SD], 8 years); married (79.3%); nuclear family (60%); basic education (62.7%); unpaid work (66.3%); and a daily household income of approximately 4 USD (61.1%).

**Conclusions:**

The caregiver burden of family caregivers of children with chronic diseases is defined and structured based on personal, family, and sociocultural factors. These features provide evidence to conduct research and implement intervention strategies with regard to families facing adversity, risk and vulnerability during a child’s disease.

## Background

Pediatric chronic diseases represent a central event that constitutes a major challenge for the family. These diseases have physical, psychological, socioeconomic, and behavioral effects on patients and their family caregivers that translate into vulnerability as well as decreased quality of life and family functioning. The international health and illness literature defines a family caregiver as a person who has a significant emotional bond with the patient. This caregiver can be a family member who forms a part of the patient’s family life cycle, offers emotional-expressive, instrumental, and tangible support, and provides assistance and comprehensive care during the chronic illness, acute illness, or disability of a child, adult, or elderly person [1].

In a longitudinal study of caregivers of husbands and wives, Zarit and colleagues proposed a useful definition of caregiver burden: the extent to which caregivers perceive that caregiving has had an adverse effect on their emotional, social, financial, physical, and spiritual functioning [2]. In addition, other authors have defined caregiver burden as a multidimensional response to the physical, psychological, emotional, social, and financial stressors associated with the caregiving experience [3]. Previous studies have shown that the lifestyles of family caregivers introduce risks to their physical, mental, and social well-being [4]. These risks derive from their daily patterns of time use that are characterized by a significant burden resulting from childcare, which increases as the child ages, from full-time parental supervision [5].

Moreover, evidence indicates that women are the main family caregivers and take responsibility for most physical tasks related to caring for children’s health [6]. Although an increase in men’s participation in assisting with care in contexts of chronic disease has been reported [7], women spend more time caring for the child in comparison with men [4]. During pediatric chronic illness, the responsibilities of the family caregiver include providing physical, psychological, spiritual and emotional support [8]. Furthermore, high levels of caregiver burden are correlated with negative outcomes for both the caregiver and child as well as risk factors for caregiver burden among mothers [9].

The empirical findings suggest that the risk factors for caregiver burden include sociodemographic and psychological aspects. Some of the first studies found that the following aspects were considered as risk factors: female sex, a low education level, residing with the care recipient, financial stress, more hours spent caregiving, a lack of choice regarding being a caregiver [10, 11], being the only caregiver of the patient since the onset of the disease, caring for a sick child for more than 1 year, caring more than 6 h a day [4–6, 12, 13], bearing a financial burden, and having unmet medical needs [14]. On the other hand, caregiver burden is characterized by psychological aspects such as high levels of burnout [15], parental stress [16], symptoms of depression [1, 17], deterioration in family functioning, symptoms of anxiety [18], negative coping styles [19], low levels of resilience [20], little social support [21], optimism [22], and effects on quality of life [23].

However, no studies are conclusive regarding the way in which the various sociodemographic and psychological aspects converge concerning the perception of overload. Likewise, the literature does not show the simultaneous influence that interpersonal issues can have on this relationship, both at the cultural and familial levels [18, 23].

Therefore, this study sought to identify multivariate sociodemographic and psychosocial variables as well as sociocultural and familial factors to analyze the caregiver burden of family caregivers of children with chronic diseases. To achieve this objective, the results of a study conducted using the Mexican population are presented that might contribute to the area of knowledge regarding the psychosocial perspectives of family caregiver adjustment and adaptation with regard to situations that involve risk, adversity, and vulnerability during a chronic disease.

## Methods

### Participants

A cross-sectional study was conducted involving 416 family caregivers of children with chronic diseases hospitalized at the National Institute of Health in Mexico City. To be included in the present study, the participants had to be over 18 years of age, be the father or mother caregiver of a child with a chronic disease, be providing care for a child with a chronic disease who required highly specialized hospital treatment at the National Institute of Health, and have read and signed an informed consent form prior to study enrollment. Potential participants who were illiterate or refused to volunteer were excluded from this study.

### Instruments

The main study variable was the caregiver burden assessed using Zarit Burden Interview [24], which was validated in the Mexican population [25]. This self-report scale evaluates respondents’ perceived tensions based on their experiences as caregivers. The instrument measures the frequency with which caregivers identify with the claims of 22 five-point Likert-type items measured using a response scale ranging from 0 (never) to 4 (always). The sum of 22 items provides a unique index of the load with a score ranging from 0 to 88. Higher scores indicate a greater level of burden. The scale explains 50% of the total variance in the construct using three factors, including effect of care on the caregiver and the caregiver-patient dyad (α = .90). These factors are also measured using five response options ranging from 0 (never) to 4 (always).

Family caregivers also responded to a battery of instruments to measure psychosocial variables (see Table 1).

**Table 1:** Description of the instruments used to characterize the psychosocial profile of family caregivers

Scales	Number of items/Response options	Factors	α
1. Family Support Questionnaire [50]	17/1 (Never) to 4 (Always)	Perception of family support	0.97
2. Social Support Network Scale [51, 52]	45/1 (Completely disagree) to 5 (Completely agree)	Friend support, family support, lack of support, religious support, and neighbor support	0.89
3. Zarit Burden Interview [24]	22/0 (Never) to 4 (Always)	Effect of care on caregiver, caregiver-patient interpersonal relationship, and self-efficacy expectations	0.90
4. Parental Stress Scale [53]	17/1 (Totally disagree) to 5 (Totally agree)	Stressors and rewards	0.89
5. Family Functioning Scale [54]	22/1 (Never) to 5 (Always)	Positive family environment, cohesion, hostility/conflict avoidance, and rules/problems expressing feelings	0.89
6. World Health Organization Well-Being Index [55]	10/0 (Never) to 3 (All the time)	Anxiety, depression, positive well-being, and coping	0.89
7. Historic-Psycho-Socio-Cultural-Premises Scale (HSCPs) [37]	33/1 (I disagree) to 2 (I agree)	Affiliative obedience, consent, self-assertion, status quo, fear of authority, Marianism and family honor	0.88

The Sociodemographic Variables Questionnaire (Q-SV) for research on family caregivers of children with chronic diseases [26] was used to collect information about the context of the family caregiver, including their age, sex, marital status, years of marriage, level of education, religion, number of children, occupation, place of residence, parental role, type of family, cycle of family life, social support networks, and monthly family income. In addition, this questionnaire included information on the sociomedical variables of the pediatric patient: sex, age, diagnosis, medical service, hospitalization time and length of time since the diagnosis of the chronic disease.

### Procedure and ethical considerations

The Ethics and Biosafety Committee of the Hospital Infantil de México Federico Gómez National Institute of Health approved the protocol of the present study under Research protocol: HIM-2013-019-SSA.1141. This study adhered to the ethical rules and considerations for research with humans currently in force in Mexico [27] as well as to those outlined by the American Psychological Association [28]. Participation in this study was voluntary. Prior to completion, participants were informed of their rights as outlined in the Helsinki Declaration [29].

All participants were provided with information regarding the study’s objective and their research rights, particularly regarding the fact that there were no consequences if they decided not to participate. Personnel trained through the Evidence-Based Medicine Research Unit at the National Institute of Health collected the data under the direction of the first author of this study. Data collection lasted approximately 5 months in 2018 and took place in the rooms of the hospitalized children and the waiting rooms of the different medical services of the institution. The researchers met with each family caregiver to provide information about the study, inform participants of their research rights, and provide them with the informed consent document. The battery of tests was administered individually.

### Data analysis

The data were analyzed using descriptive statistics and Student’s *t*-test for the psychosocial variables based on the sex of the caregiver. Multivariate analysis was performed through hierarchical linear regression models to observe predictive relationships between the four groups of variables (sociodemographic, psychological, family, and sociocultural) and the dependent variable, caregiver burden, in women and men caring for children with chronic diseases. The program SPSS version 24 was used for all analyses.

## Results

### Descriptive analysis of the sociodemographic characteristics of the children

With regard to the sociodemographic characteristics and sociomedical variables of children with chronic illnesses, the following information was obtained: 47.4% of the patients were girls, and 52.6% were boys; the mean age of the children with chronic diseases was 5.91 years, and the standard deviation (SD) was 5.03; most children had been hospitalized for a week (63.2%), followed by those who had been there for a month (21.6%), six months (8.2%) and more than six months (7%); 37.3% of the patients had been diagnosed up to three months prior to data collection, 30.3% had been diagnosed between four months and one year prior, and 32.4% had been diagnosed three or more years prior to the study; the diagnosis for most patients (74%) was some type of cancer (e.g., leukemia, tumors, neuroblastoma), while 26% suffered from other chronic diseases (e.g., asthma, terminal chronic renal failure, nephrotic syndrome).

### Sociodemographic profile of mothers and fathers who care for children with chronic diseases

The results showed a greater percentage of female family caregivers (81.7%) than male family caregivers (18.3%) in the total number of families interviewed. Table 2 presents the sociodemographic characteristics of the participants. There are similarities between the profiles of women and men because, on average, they were young (31 years old for women and 34 years old for men), lived with their romantic partners (76.5% of women and 92.1% of men), had a basic level of education (62.6% of women and 63.1% of men), count on their family as their main support network (87.1% of women and 69.7% of men), and typically earned a monthly family income equivalent to 100 United States dollars (USD) in the case of women (63.5%) and 150 USD in the case of men (50%). The variables with different response trends according to the sex of the family caregiver were (a) occupation, with 80.9% of women having unpaid work and 75% of men having a paid job, and (b) nuclear family (46.2% of women and 72.4% of men).

**Table 2:** Sociodemographic characteristics of the 416 family caregivers: men and women

Sociodemographic variables	Women (*n* = 340)		Men (*n* = 76)	
	Mean (SD*)	No. (%)	Mean (SD^a^)	No. (%)
Age	31.05 (7.74)		34.43 (8.71)	
Marital status				
Married		260 (76.47)		70 (92.10)
Single		80 (23.53)		6 (7.90)
Education				
Basic		213 (62.65)		48 (63.15)
Middle		87 (25.59)		21 (27.63)
Higher		29 (8.53)		6 (7.90)
None		11 (3.23)		1 (1.32)
Profession				
Unpaid work (exclusively devoted to home or studies)		275 (80.88)		2 (2.63)
Paid work		54 (15.88)		57 (75)
Unemployed		11 (3.24)		17 (22.37)
Type of family				
Nuclear		157 (46.17)		55 (72.36)
Semi-extended family		58 (17.05)		8 (10.52)
Extended		37 (10.88)		6 (7.90)
Single parent		63 (18.52)		2 (2.64)
Other		25 (7.38)		5 (6.58)
Support networks				
Family		296 (87.05)		53 (69.73)
Institutions/government/friends		44 (12.95)		23 (30.27)
Monthly family income				
Between USD 120 and USD 160		216 (63.53)		38 (50)
Between USD 161 and USD 350		114 (33.53)		34 (44.73)
Between USD 351 and USD 520		9 (2.65)		4 (5.27)
Between USD 521 and USD 800		1 (.29)		–

^a^*SD* Standard deviation

### Psychosocial profile of mothers and fathers who care for children with chronic diseases

The results indicated the existence of a similar psychosocial profile for men and women because Student’s *t*-test showed no significant differences in any of the comparison variables. Thus, according to the scores obtained, no significant differences were found in the group of psychological, family, and sociocultural variables based on the sex of the family caregivers. See Table 3.

**Table 3:** Scores obtained for the psychosocial variables by family caregivers: men and women

Psychosocial variables	Possible value range	Minimum-maximum	Mean (SD)	Minimum-maximum	Mean (SD)	t	p
		Women (*n* = 340)		Men (*n* = 76)			
Sociocultural factors							
Sociocultural premises	33–66	33–64	49.30 (6.28)	38–62	50.21 (5.54)	1.15	0.24
Social support networks	4–225	82–192	159.63 (18.01)	109–213	156.23 (19.75)	−1.47	0.14
Family factors							
Family support	17–68	17–68	59.50 (10.28)	34–68	59.21 (9.54)	−.22	0.82
Family functioning	22–110	35–106	86.82 (12.17)	59–105	84.43 (11.21)	−1.56	0.11
Psychological factors							
Well-being	0–30	3–30	18.13 (5.12)	9–30	18.55 (5.51)	0.64	0.51
Stressors	7–35	7–32	12.72 (5.48)	7–26	12.89 (5.16)	0.25	0.80
Anxiety	0–63	0–62	14.74 (12.83)	0–63	13.56 (12.90)	0.75	0.47
Caregiver burden	0–88	0–88	23.12 (12.16)	1–66	23.12 (12.16)	0.36	0.71

With regard to sociocultural factors, female caregivers scored 49 points on average for the variable “agreement with sociocultural premises,” whereas male caregivers scored 50, with a range of 33 to 66 points. This indicates that in the sample studied, this attribute was manifested in a moderate way. With regard to social support networks, on average, women obtained 160 points, and men obtained 156 out of a maximum of 225. This finding implies that mothers and fathers have a moderately high perception of access to social support networks.

In relation to specific family support, women and men reported an average of 59 points, within a range of 17 to 68, suggesting that caregivers perceive a high level of support from their families. There is also a high perception of family functioning; on average, women scored 87 points, and men scored 84, out of a maximum of 110 points. Family functioning refers to a positive family environment, cohesion, less hostility/conflict avoidance and fewer rules/problems when expressing feelings.

Regarding psychological factors, the average scores reported by female and male caregivers were 18.13 and 18.55, respectively, with a range of 0 to 30 points. Thus, men and women reported moderate scores. The identification of stressors was low to moderate for the total sample of caregivers because the averages were close to 13 out of the 35 points that could be obtained. Caregivers’ perception of burden and anxiety was low in women and in men. The averages observed were around the 25th percentile.

To analyze the multivariate relationships between the psychosocial and sociodemographic profile and the burden perceived by caregivers, a hierarchical multiple regression model was estimated. The first block of variables included in the model comprised the following sociodemographic indicators: age of the caregiver, having upper secondary education or higher (dummy variable) and being female (dummy variable). The second set of variables entered into the model consisted of sociocultural factors: historical sociocultural premises and social support networks. The third set of variables entered into the equation included family factors: family support and family functioning. The fourth and final step considered psychological variables: perception of well-being, stress and anxiety.

Table 4 shows the results of the analysis of the hierarchical multiple regression model. The four blocks of variables—sociodemographic, sociocultural, family and psychological—had a significant predictive ability on the dependent variable of caregiver burden, and together they explained 40% of its variance [*Adjusted R*^*2*^ = 0.40; *F*(3, 40) = 59.90, *p <* 0.01]. With the exceptions of age and sex of the caregiver, all variables that were included in the model exhibited a significant relationship. Positive predictors of caregiver burden included historical sociocultural premises (β = 0.14, *p <* 0.01), stressors (β = 0.33, *p <* 0.01) and anxiety (β = 0.32, *p <* 0.01). Negative predictors included upper middle education or higher (β = − 0.13, *p <* 0.01), social support networks (β = −.19, *p <* 0.01), family support (β = − 0.11, *p <* 0.05), family functioning (β = − 0.26, *p <* 0.01) and well-being (β = − 0.10, *p <* 0.05).

**Table 4:** Hierarchical linear regression model of the perceived burden for family caregivers (*n* = 416)

Variables	Correlation with DV	B	β	F (df)	R and R^2^ values
Sociodemographic					
Higher education	−0.12	−3,38	−0.13**	2.89 (3, 41)*	R = 0.14
Age of caregiver	−0.04	− 0.09	− 0.06		R^2^ = 0.02
Female caregiver	− 0.01	− 0.94	− 0.03		R^2^fit = 0.01
					R^2^change = 0.02*
Sociocultural					
Sociocultural historical premises	0.15	0.28	0.14**	10.88 (2, 40)***	R = 0.27
					R^2^ = 0.07
					R^2^fit = 0.06
Social support networks	−0.20	−0.13	−0.19***		R^2^change = 0.05***
Familiar					
Family support	−0.28	−0.14	− 0.11*	19.22 (2, 40)***	R = 0.39
					R^2^ = 0.15
					R^2^fit = 0.14
Family functioning	−0.36	−0.26	−0.26***		R^2^change = 0.08***
Psychological					
Well-being	−0.38	−0.24	− 0.10*	59.90 (3, 40)***	R = 0.64
					R^2^ = 0.41
Stressors	0.48	0.74	0.33***		R^2^fit = 0.40
Anxiety	0.48	0.30	0.32***		R^2^change = 0.26***

DV = dependent variable; * *p* ≤ 0.05; ** *p* ≤ 0.01; ***p ≤ 0.001

## Discussion

The aims of this study were to identify the relationship between sociodemographic and psychosocial factors, to examine the variables that predict caregiver burden based on psychological, family, sociocultural and sociodemographic factors in family caregivers of children with chronic diseases. The study’s results characterized mothers and fathers in their role as caregivers for their preschool- and school-aged children who were hospitalized and under treatment for cancer or another chronic disease in the National Institute of Health in Mexico City.

With regard to the sociodemographic factors, most cases involved married mothers with basic schooling who were homemakers, lived in a nuclear family, and had low income and whose main support network was the family. These results are consistent with the profile identified in Latin America and the Caribbean, where long-term care represents a type of unpaid work performed by women, as required by the multiplicity of psychological and family demands [30, 31]. In addition, the profile that defines the families of children with chronic conditions is characterized by adversity associated with the diagnosis, as well as social vulnerability and psychosocial risk during the disease and long-term treatment [4–6, 10, 32].

Socioeconomic conditions are permeated by sociocultural influences that help to explain, for example, the prevalence of women who undertake this role. In particular, Mexican women play an active role in the care of sick family members [33]. A series of historical sociocultural premises underlies this pattern, which highlights affection, sacrifice and self-denial as typically female attributes that predispose women toward care [1, 34].

Although more women than men sought out this role, the results of this study indicate that there are no differences in the burden of care, well-being, anxiety nor stressors perceived by mothers and fathers. The same is true for support mechanisms in the family dynamics of the caregiver. Even in the multivariate analysis of the sociodemographic and psychosocial aspects of caregiver burden, the sex of the caregiver was not a significant predictor. One possible interpretation of this fact is that although it is more common for women to look after a sick child, by assuming this role, both parents are presented with similar experiences and challenges, and the different adaptive results are related to psychosocial aspects more than to demographic features per se [35].

In this regard, it should be noted that although the variables analyzed in this study as part of the psychosocial and sociodemographic profile together explained 40% of the variance of the burden in caregivers, the first block of variables showed a minor contribution to the total (2.1%) in comparison with the blocks related to sociocultural (5.4%), family (8%) and psychological (26%) variables. This is an important finding because it describes the simultaneous role of the profiles analyzed (sociodemographic and psychosocial) while pointing to the differences in the magnitudes of these relationships. Based on the above findings, further multicultural and multivariate analysis is suggested to advance the understanding of the social, cultural, family and psychological factors that characterize the process of adaptation of individuals and their families [35, 36].

Regarding the psychosocial characteristics, the two variables in the sociocultural variable block showed a significant contribution to the explanation of caregiver burden. Following historical sociocultural premises increases the perceived burden. This may be due to the conflict situation that is currently experienced by families in our society, where certain aspects that characterize traditional families, including the importance of child obedience, the protection of women and family honor, coexist with traits of families in transition who retain some features from the past, such as fear of authority, but who try to change others, such as the search for autonomy and independence [37]. On the other hand, access to social support networks decreases caregiver burden. This finding confirms that the existence of social support networks promotes psychological health and reduces the psychosocial effects and consequences of care during chronic diseases [1, 10, 38, 39].

With respect to family variables, it was also found that the two indicators analyzed contributed individually and significantly to the explanation of the burden on caregivers. Family functioning focused on a positive family environment, family cohesion and less hostility, conflicts and problems in the expression of feelings, which is associated with lower perceived burden. Similarly, the perception of greater social support from the family contributes to a lower perception of burden. These results are consistent with previous studies that have emphasized the central role of the family in Mexican culture [37, 40, 41], the influence of the dynamics of intrafamily relationships during care [42] and the importance of family support on the well-being of the caregiver [43].

The psychological variables block greatly affected the total variance in caregiver burden. The three variables considered were significant predictors, indicating that stressors and anxiety are positively associated with burden, while well-being has an inverse relationship with burden. These results are consistent with the extensive literature that describes the relationships among stress, anxiety and exhaustion in parents of children with chronic diseases and how these factors damage parents’ well-being [31, 35, 44].

It should be noted, however, that the perception of burden and anxiety tended to be low in the sample analyzed because the averages observed were around the 25th percentile. Similarly, scores related to child upbringing ranged from low to moderate. These results contrast with previous empirical findings that reported consequences of care and negative effects on the psychological health of family caregivers of pediatric patients [14, 21, 45–47]. The reason for this discrepancy, in addition to the seemingly protective role of psychosocial resources on caregivers’ well-being in this study, could be that more than 60% of the cases had only a week of hospitalization at the time of data collection. Thus, the amount of time spent in this critical situation may influence the level of burnout experienced by caregivers. Another striking feature of the sample studied is that mothers and fathers were taking care of their children in all cases, so the type and quality of the parental link could exert a moderating effect on caregivers’ adaptive results. Future studies comparing different family ties (e.g., grandmothers, aunts, uncles) and levels of attachment could help to confirm this hypothesis [36]. Similarly, longitudinal research designs could identify the effects of time on the experience of care [48].

The practical implications of this study suggest that the theoretical, practical, social and methodological importance of obtaining the profile of caregivers to account for both psychosocial and sociodemographic dimensions substantially contributes to research on families of children with chronic diseases by helping to generate measurement, assessment and intervention programs to reduce the impact of the disease, its psychosocial effects, the consequences of care and caregiver burnout [44, 49].

## Conclusions

Previous studies have emphasized the importance of studying the impact of sociodemographic and psychosocial variables on the role of family caregivers and the latter’s adjustment to the disease and treatment of their children. However, most studies address the two perspectives separately, thus offering intervention alternatives that are not comprehensive. This research offers an interesting perspective by presenting a comprehensive approach to the sociodemographic and psychosocial factors that constitute the profile of the caregiver in contexts of adversity resulting from pediatric disease. In this sense, the characterization of the family caregiver results from the continual interaction among psychological, sociocultural and family factors and the strength required to confront and overcome the disease. One of the strengths with the greatest positive impact on the caregiver is family support, which contributes to the process of positive adaptation during the diagnosis and long-term treatment of the child.

## Abbreviations

DV: dependent variable
SD: Standard deviation
USD: United States dollars
